# Utilization of maternal healthcare services in women experiencing spousal violence in Pakistan: A comparative analysis of 2012-13 and 2017-18 Pakistan Demographic Health Surveys

**DOI:** 10.1371/journal.pone.0239722

**Published:** 2020-09-25

**Authors:** Sehar-un-Nisa Hassan, Erum Memon, Mala Shahab, Sarwat Mumtaz

**Affiliations:** 1 College of Public Health and Health Informatics, University of Ha’il, Ha’il, Kingdom of Saudi Arabia; 2 Department of Gynecology and Obstetrics, Jinnah Medical and Dental College, JSMU University, Karachi, Pakistan; Xi'an Jiaotong University School of Medicine, CHINA

## Abstract

**Background:**

Pakistan and other developing countries need to address disparities in maternal health care and factors associated with it. This justifies tracking the progress on two important indicators ‘spousal violence’ and ‘maternal health care utilization’ to improve maternal health and achieve Sustainable Development Goals (SDGs) for these nations.

**Objective:**

The objective of this study is to compare the data from the latest two Demographic Health Surveys of Pakistan to identify trends in prevalence of various forms of spousal violence and maternal healthcare utilization and to determine the predictive role of spousal violence in poor maternal health.

**Methods:**

We conducted a retrospective analysis of nationally representative data from the 2012–13 and 2017–18 PDHS. The data used in this analysis is from the domestic violence module and core women’s questionnaire. Spousal violence and sociodemographic background were predictor variables. Terminated pregnancy, number of pregnancy losses, number of antenatal visits for last birth and institutional delivery for last birth were taken as indicators of maternal health. Logistic regression analysis was conducted to test for association between maternal health indicators and various forms of spousal violence after controlling for sociodemographic variables.

**Results:**

Almost one quarter of women experienced physical and emotional violence as revealed by both surveys. Binary analysis revealed that all forms of spousal violence significantly associate with maternal health variables in both surveys. The comparison of results on logistic regression analysis showed that odd ratios were relatively higher for 2012–13 as compared to 2017–18 PDHS. Logistic regression analysis from 2017–18 data showed that experience of less severe physical violence (OR = 1.26; 95% CI, 1.08–1.47), severe physical violence (OR = 1.41; 95% CI, 1.09–1.83), sexual violence (OR = 1.39; 95% CI, 1.02–1.89), physical violence during pregnancy (OR = 1.37; 95% CI, 1.07–1.76) augment the risk of terminated pregnancy. Emotional violence decreases the likelihood for institutional delivery (OR = 0.64; 95% CI, 0.45–0.93) and above than four antenatal visits (OR = 0.54; 95% CI, 0.37–0.79).

**Conclusions:**

Strategies to prevent spousal violence should be at the core of maternal health programs because health sector provides a platform to challenge social norms and promote attitudes that disapprove spousal violence which are essential for promoting gender equality, women empowerment (SDG 3) and improve maternal health (SDG 5).

## Introduction

Maternal health is a well-recognized aspect of women’s health. Women in developing countries more commonly suffer from pregnancy related complications, premature delivery of children and maternal death. This gap is as high as up to 33 percent between developed and developing countries [1]. Pakistan still lapses behind on maternal health indicators from many other countries of the world [2]. Some of the risk factors are malnutrition, limited access to good quality healthcare services, preference for traditional methods of treatment rather than seeking medical aid at time of delivery, short birth intervals, poor knowledge about available services and late antenatal booking [3, 4]. Literature reported that 78% of maternal death are due to direct causes such as hemorrhage, sepsis, eclampsia, rupture of the uterus, and abortions [5]. Other than medical complications, it is important to investigate predictive role of other factors such as experience of spousal violence in intimate relations on women gynecological health [6].

Women in both developed and developing countries face various forms of violence among which spousal violence has emerged as a significant social nuisance and public health problem. World Health Organization (WHO) [7], defined spousal violence as “a behavior within an intimate relationship that causes physical, sexual or psychological harm, including acts of physical aggression, sexual coercion, psychological abuse and controlling behaviors”. The global prevalence of spousal violence is reported through multi-country study lie in range of 23–30% [8]. Review of statistics on prevalence of spousal violence from Pakistan [9] and other developing countries [10, 11] have shown that even larger percentage (34–43%) of women experienced physical, psychological and sexual forms of violence in their everyday lives.

In this context, it is imperative to recognize that spousal violence is a major human rights issue but also a significant determinant of women’s physical, reproductive, maternal and emotional health [12]. The impacts of spousal violence on women’s acute and chronic health conditions are reported in literature [13]. Findings revealed that women are presented with diverse nature of medical complications due to spousal violence that range from minor to major physical injuries [14], poor sexual, reproductive and maternal health [15] as well as psychological conditions such as depression, anxiety and post-traumatic stress disorder (PTSD) [16]. The negative impacts of spousal violence are not limited to women’s health but children in these families are also vulnerable to bear physical and mental health consequences of such adverse experiences [17].

Women in developing countries frequently experience emotional violence, economic control as well as physical and sexual violence from their male partners. According to global and regional estimates on prevalence of Intimate Partner Violence (IPV), 30 percent of ever-partnered women at some point in their lives have experienced physical and/or sexual violence by an intimate partner and at least 10 percent of them faced IPV during their pregnancy [18]. Women in developing countries have little control over their reproductive lives [19]. They are given less choice to use contraceptive methods which impacts women’s reproductive health [20]. Previous studies have shown that in Pakistan 18% of the pregnant women are more likely to have anxiety and/or depression, linked with physical or sexual and verbal abuse during pregnancy [21].

Studies from other developing countries of South Asian region, such as in-depth analysis of data from 2005–2006 India’s National Family Health Survey reported that almost 12% of women experienced severe physical violence and 14% of women had less severe injuries due to spousal violence [22]. An analysis of data from Bangladesh Demographic Health Survey showed that (53%) of mothers reported physical or sexual violence from spouse in the year prior to the survey [23]. In developing countries, factors such as less education, low socioeconomic status, living in rural areas, big family size, drinking or alcohol abuse by husband, jealousy, suspicion and husband’s need to keep control increases the woman’s risk for spousal violence [24]. Review of literature from Pakistan and other countries supported evidence about negative impacts of violence on women physical, mental, reproductive and sexual health [25, 26].

In developing countries, spousal violence is rooted in a wider context of gender inequality. Gender-based violence and discrimination are major contributing factors of slow progress towards sustainable developmental goals as indicated by the latest rankings on Gender inequality index for Pakistan, which is 148 out of 149 countries [27]. Despite such alarming statistics, there are limited evidence about predictive role spousal violence on maternal health indicators from this part of world. This gap is a major obstacle to devise appropriate public health policies, maternal health programs and social interventions in context of developing countries.

This study aims to address this gap by doing analysis from the last two Pakistan Demographic Health Surveys conducted in four major provinces and capital city completed in years 2012–13 and 2017–18.

### Objectives of the research

This study has three objectives.

1. To report the trends in prevalence of various forms of spousal violence and maternal health indicators.
2. To estimate the association between experience of various forms of spousal violence and maternal health.
3. To determine the predictive role of various forms of spousal violence with maternal health.

## Methods

### Data

This study draws on data from the last two Pakistan Demographic and Health Survey (PDHS) 2012–13 and 17–18 available at https://dhsprogram.com. Demographic Health Surveys (DHS) are the biggest household surveys conducted four times in Pakistan and contains rich information on demography and maternal health variables for ever-married women aged (15–49 years). For the main survey, a nationally representative sample was obtained with a two-stage, stratified random sampling design. We selected these two surveys because spousal violence was investigated first time on a sub-sample in 2012–13 PDHS survey. In previous surveys of DHS in Pakistan, experience of spousal violence was not included. The PDHS 2012–13 obtained data about physical and emotional forms of spousal violence only. PDHS 2017–18 included data on experience of (physical, emotional and sexual) forms of spousal violence. The main survey comprises of larger sample size (N = 13,558) in PDHS 12–13 survey [28] and (N = 14,161) in PDHS 17–18 survey [29]. Domestic violence module is administered on sub-sample of women and therefore, this study restricted its analysis to a sub-sample (N = 3,687) women from PDHS 2012–13 survey and (N = 4,085) from PDHS 2017–18 survey. In this analysis, variables on spousal violence included experience of less severe physical violence, severe physical violence, emotional violence sexual violence, physical violence during pregnancy. Data on maternal health variables included, terminated pregnancy, institutional delivery and number of antenatal visits for the most recent birth.

### Measures

#### Predictor variables

The predictor variable in this analysis is spousal violence, assessed through items on domestic violence module. In both demographic health surveys (PDHS 2012–13 and PDHS 2017–18) experience of spousal physical violence, emotional violence and physical violence during pregnancy were inquired from ever-married women. Intensity of physical spousal violence was coded as “Experience of any severe violence” and “Experience of any less severe violence”. The experience of sexual violence in spousal relationship was assessed for the first time in PDHS-2017-18 survey. On each indicator of spousal violence, variable is coded as “1” if yes and “0” if no experience of these forms of spousal violence.

#### Control variables

In the literature, numerous demographic and social variables have been shown to influence maternal health of women in developing countries [30–32]. The potential influence of confounding variables is controlled in regression analyses. These included women age, women education, work status, husband education, husband job status, place of residence and wealth index. The descriptive analysis on these variables are presented in Table 1.

**Table 1:** Demographic characteristics of respondents who participated in PHDS 2012–13 and PDHS 2017–18.

Sociodemographic Characteristics	PDHS 2012–13 (N = 3687)		PDHS 2017–18 (N = 4085)	
	Frequency	%	Frequency	%
**Women age (years)**				
15–19	116	3.1	146	3.6
20–24	478	13.0	555	13.6
25–29	691	18.7	797	19.5
30–34	693	18.8	816	20.1
35–39	699	19.0	797	19.5
40–44	524	14.2	512	12.5
45–49	486	13.2	462	11.3
**Education**				
No education	2051	55.61	2087	51
Primary	530	14.4	565	13.8
Secondary	654	17.7	818	20.0
Higher	452	12.3	615	15.1
Women Current employment status*				
Unemployed	2864	77.7	3514	86.0
Employed	817	22.2	571	14.0
Husband Education*				
No education	1149	31.2	1112	27.2
Primary	498	13.5	538	13.2
Secondary	1165	31.6	1348	33
Higher	867	23.5	964	23.6
**Husband Current employment status**				
Unemployed	115	3.1	195	4.8
Employed	3572	96.7	3890	95.2
Wealth Index**				
Poorest	683	18.5	794	19.4
Poorer	715	19.4	943	23.1
Middle	684	18.6	787	19.3
Richer	768	20.8	747	18.3
Richest	837	22.7	814	19.9
**Place of Residence**				
Urban	1734	47.0	1978	48.4
Rural	1953	53.0	2107	51.6

*The total sample size may vary because of missing values on some demographic variables.

** Households are given scores based on the number and kinds of consumer goods they own, ranging from a television to a bicycle or car, and housing characteristics such as source of drinking water, toilet facilities, and flooring materials. These scores are derived using principal component analysis. National wealth quintiles are compiled by assigning the household score to each usual (de jure) household member, ranking each person in the household population by their score, and then dividing the distribution into five equal categories, each with 20% of the population

#### Outcome variables

*Institutional delivery* is defined as delivery in a healthcare facility including (government or private hospital, clinic, basic health unit, rural health center, community midwife set up) [28, 29]. This variable is coded as “1” if a woman has delivery in any of this institution for last childbirth and “0” if it was in ‘home’.

*Antenatal care* is defined as having a minimum of four antenatal visits to skilled health workers for last birth. This was the minimum requirement for maternal health care as per previous guidelines and as per recent recommendations by World Health Organization (WHO) there should be a minimum of eight contacts [33]. This variable is coded as “1” if a woman has at least four antenatal visits for last birth and “0” if less than four antenatal visits for last birth.

*Ever had terminated pregnancy* is defined as woman ever having a pregnancy that resulted in miscarriage, abortion, or stillbirth. This variable is coded as “1” if a woman has one or more terminated pregnancy under the definition and “0” if otherwise.

*Number of pregnancy losses* is defined as number of times woman having a pregnancy that resulted in miscarriage, abortion, or stillbirth. This variable is coded as “0” if a woman has no terminated pregnancy, “1” if a woman has one terminated pregnancy and “2” for two terminated pregnancies and “3 & above” for three and more than three terminated pregnancies.

### Statistical analysis

Descriptive statistics are employed to describe characteristics on all variables. Associations between various forms of spousal violence and maternal health indicators were determined by using chi-square tests. We used a logistic regression model to assess the impact of various forms of spousal violence on maternal health of women by calculating adjusted odds ratios (ORs) with 95% confidence intervals (CIs) while controlling for confounding variables. The statistical analyses for this study were performed using SPSS IBM.

### Ethical approval

The study used open-access dataset of PDHS 2012–13 and 2017–18. The survey protocol was reviewed and approved by the National Bioethics Committee, Pakistan Health Research Council, and ICF Institutional Review Board [28, 29].

## Results

### Demographic characteristics and living conditions of participants

The PDHS 2012–13 and PDHS 2017–18 obtained data about experience of spousal violence on Domestic Violence Module from a sub-sample of 3,687 and 4,085 currently married women aged 15–49 years respectively. 51–56% of the women in this sub-sample had not obtained formal education and 27–31% of participants’ husbands had not obtained formal education. 77–86% of the women were not involved any paid work and 3–4% of participant’s husbands were unemployed. 47–48% of the participants were living in urban regions and 51–53% were living in rural regions. (Table 1). This presentation of demographic characteristics appropriately presents demographic profile of Pakistan population.

### Experience of spousal violence

Table 2 shows frequency and percentages of women’s experiences of various forms of spousal violence. Substantial percentage of women reported experiencing spousal violence in both surveys, however, there is declining trend in terms of prevalence of less severe physical violence, which was 28.4% in 2012–13 survey and decreased to 24.4% in 2017–18 survey. There are no differences in rates of emotional violence in both surveys. The prevalence rate of sexual violence is 4.5% as per 2017–18 PDHS (Table 2).

**Table 2:** Distribution of experience of various forms of spousal violence.

Women’s Experience of Spousal Violence Variables	PDHS 2012–13 (N = 3687)		PDHS 2017–18 (N = 4085)	
	Frequency	%	Frequency	%
Experienced Less Severe Physical Violence	1033	28.4	998	24.4
Experienced Severe Physical Violence	257	7.0	280	6.9
Experienced Emotional Violence	1154	31.3	1243	30.4
Experienced Sexual Violence	*-	*-	184	4.5
Experienced physical violence during pregnancy	391	10.6	291	7.6

*No data obtained about experience of sexual violence in 2012–13 survey

### Association of various forms of spousal violence with maternal health

Findings based on comparison of PDHS 2012–13 and PDHS 2017–18 showed, there is little decline in rates of spousal violence and slight improvement on maternal health conditions of participants across the years between these two surveys. In 2012–13 PDHS survey 28% of women reported less severe physical violence where as in 2017–18 PDHS survey it was declined to 24% and in 2012–13 survey 10.6% of women reported physical violence during pregnancy which was declined to 7.6% in 2017–18 survey. In 2012–13 PDHS survey 60% of women reported less than four antenatal visits where as in 2017–18 PDHS survey it was declined to 51%. Similarly, 46% women reported no institutional delivery in 2012–13 survey which was 33% in 2017–18 survey. The rates of terminated pregnancy in 2012–13 survey was approximately 36%, which was lowered to 33% in 2017–18 survey. The dataset from both surveys showed that significantly higher proportions of women who experienced spousal violence had less than four antenatal visits, no institutional delivery and terminated pregnancy (Table 3). Findings based on analysis of PDHS 2012–13 dataset demonstrate that both physical and emotional violence significantly associate with poor antenatal care at (p < .001), decreased likelihood of institutional delivery at (p < .001) and high risk of terminated pregnancy at (p < .01) (Table 3). The analysis of PDHS 2017–18 dataset demonstrated that the experience of sexual violence and violence during pregnancy significantly associate with less than four antenatal visits and terminated pregnancy at (p < .05) (Table 3). Experience of physical and emotional violence significantly associate with less than four antenatal visits and non-institutional delivery at (p < .001). (Table 3).

**Table 3:** Distribution recent birth and ever had terminated pregnancy of less than 4 ANC visits for most recent birth, institutional delivery for most recent birth.

	Outcome variables											
	Less than 4 ANC visits for participants				Participant had No Institutional Delivery for the most recent childbirth				Participant Ever had terminated pregnancy			
(n) after including valid cases in analysis*	PDHS 2012–13 (n = 2038)		PDHS 2017–18 (N = 2255)		PDHS 2012–13 (n = 2038)		PDHS 2017–18 (N = 2261)		PDHS 2012–13 (N = 3687)		PDHS 2017–18 (N = 4085)	
	*f*	%	*f*	%	*f*	%	*f*	%	*f*	%	*f*	%
	1225	60.0	1157	51.3	939	46.1	752	33.2	1325	35.9	1313	32.1
Experienced Less Severe Violence*	χ2 = 53.02, df = 1, p < .001		χ2 = 35.78, df = 1, p < .001		χ2 = 50.02, df = 1, p < .001		χ2 = 18.64, df = 1, p < .001		χ2 = 24.43, df = 1, p < .001		χ2 = 10.29, df = 1, p < .01.	
No	777	54.9	771	47.4	579	40.9	499	30.6	889	33.5	951	30.8
Yes	448	72.0	386	61.5	360	58.0	253	40.0	436	42.2	362	36.3
Experienced Severe Violence*	χ2 = 12.21, df = 1, p < .001		χ2 = 16.88, df = 1, p < .001		χ2 = 10.86, df = 1, p < .01		χ2 = 11.1, df = 1, p < .001		χ2 = 10.13, df = 1, p < .01		χ2 = 8.54, df = 1, p < .01	
No	1114	59.0	1051	50.1	850	45.1	680	32.4	1209	35.3	1200	31.5
Yes	111	73.5	106	67.1	89	58.9	72	45.3	116	45.1	113	40.0
**Experienced Emotional Violence**	χ2 = 40.11, df = 1, p < .001		χ2 = 19.39, df = 1, p < .001		χ2 = 52.94, df = 1, p < .001		χ2 = 10.73, df = 1, p < .001		χ2 = 16.80, df = 1, p < .001		NS	
No	749	55.2	724	48.0	548	40.4	468	31.0	854	33.7	892	31.4
Yes	476	69.8	433	57.9	391	57.1	284	37.9	471	40.7	421	33.9
**Experienced Sexual Violence**	*-		χ2 = 3.98, df = 1, p < .05		*-		NS		*-		χ2 = 5.00, df = 1, p < .05.	
No	*-	*-	1092	50.9	*-	*-	713	33.1	*-	*-	1240	31.8
Yes	*-	*-	65	60.7	*-	*-	39	36.4	*-	*-	73	39.7
**Experienced physical violence during pregnancy**	χ2 = 34.15, df = 1, p < .001		χ2 = 9.20, df = 1, p < .05		χ2 = 31.9, df = 1, p < .001		NS		χ2 = 7.63, df = 1, p < .01		χ2 = 6.19, df = 1, p < .05.	
No	1031	57.7	1044	50.4	781	43.7	683	32.9	1149	37.5	1192	34.0
Yes	194	77.0	113	62.1	158	62.7	69	37.6	176	44.8	121	41.2

* refer to physical acts of violence; *(ANC = antenatal care). NS = Non-significant

* No data obtained about experience of sexual violence in 2012–13 survey

*Valid cases refer to the total number of subjects whose data is complete on variables in analysis.

### Impact of spousal violence on maternal health

A summary of odd ratios [Exp(B)] with a 95% confidence interval are given in Table 4 after adjusting for demographic variables. Experiencing ‘severe violence’ emerged as the strongest predictor of ‘terminated pregnancy’. The odds of terminated pregnancy were 1.4 times higher (p < .01) for those women who experienced severe physical violence. Findings also demonstrate that experience of spousal violence increases the risk for more number of terminated pregnancy. The odds of 2 & above pregnancy losses is 1.7 times higher (p < .01) for those women who experienced severe physical violence. Both sexual violence and physical violence during pregnancy significantly (p < .05) predict terminated pregnancy and 2 & above pregnancy losses.

**Table 4:** Adjusted odd ratios (OR)s and 95% CIs for the impact of spousal violence on maternal health indicators.

	PDHS 2012–13					PDHS 2017–18				
	(N = 3687)					(N = 4085)				
	OR (95%CI)					OR (95%CI)				
	Outcome Variables					Outcome Variables				
	Ever had terminated pregnancy No = (Ref)	No of pregnancy losses 0 = (Ref)		Institutional Delivery for last birth Yes = (Ref)	Less than four ANC visits No = (Ref)	Ever had terminated pregnancy No = (Ref)	No of pregnancy losses 0 = (Ref)		Institutional Delivery for last birth Yes = (Ref)	Less than four ANC Visits No = (Ref)
		1	2 & above				1	2 & above		
**Less Severe Violence**	1.44*** (1.24–1.68)	1.43*** (1.19–1.72)	1.52** (1.19–1.95)	0.61 (0.49–0.74)	0.60*** (0.47–0.76)	1.26** (1.08–1.47)	1.24* (1.03–1.49)	1.38* (1.06–1.79)	0.87 (0.71–0.97)	0.76* (0.61–0.94)
**Severe violence**	1.49*** (1.15–1.94)	1.39* (1.02–1.91)	1.58** (1.05–2.36)	0.68* (0.47–0.99)	0.64* (0.42–0.98)	1.41** (1.09–1.83)	1.41* (1.04–1.91)	1.73** (1.15–2.59)	0.64** (0.45–0.92)	0.54** (0.37–0.79)
**Emotional violence**	1.31*** (1.12–1.51)	1.28** (1.07–1.53)	1.26* (0.99–1.61)	0.56** (0.45–0.69)	0.63** (0.50-.079)	1.07 (0.93–1.24)	1.12 (0.94–1.33)	1.13 (0.88–1.46)	0.93* (0.76–0.98)	0.86 (0.70–1.06)
**Sexual violence**	-*	-*		-*	-*-*	1.39* (1.02–1.89)	1.54* (1.08–2.18)	1.07 (0.81–1.07)	0.93 (0.60–0.94)	0.71 (0.45–1.10)
**Physical violence during pregnancy**	1.35* (1.08–1.67)	1.16 (0.89–1.52)	1.61** (1.16–2.24)	0.58*** (0.43–0.78)	0.54** (0.38–0.72)	1.37* (1.07–1.76)	1.41* (1.06–1.89)	1.39* (0.91–2.12)	0.99 (0.71–0.93)	0.75 (0.53–1.06)

* refer to physical acts of violence; (ANC = antenatal care).

-* No data obtained about experience of sexual violence in 2012–13 survey

***p < .001;

**p < .01; *p < .05 **Adjusted odds ratios were **s**imultaneously adjusted for all sociodemographic variables

Table 4 demonstrate that data analysis of PDHS 2012–13 depicts all forms of spousal violence significantly decrease the likelihood of institutional delivery for the last birth. However, in PDHS 2017–18 dataset ‘severe physical violence’ and ‘emotional violence’ significantly decrease the likelihood of institutional delivery for the last birth with (OR = 0.64; 95% CI, 0.45–0.92) and (OR = 0.93; 95% CI, 0.76–0.98) respectively.

Spousal violence also negatively influences antenatal care. The findings from PDHS 2012–13 shows that all forms of spousal violence significantly decrease the likelihood of more than four antenatal visits for the last birth. In PDHS 2017–18 dataset, ‘less severe physical violence’ and ‘severe physical violence’ significantly decrease the likelihood of more than four antenatal visits for the last birth with (OR = 0.76; 95% CI, 0.61–0.94) and (OR = 0.54; 95% CI, 0.37–0.79) respectively (Table 4).

Tables 1 and 2 in (in the S1 Appendix online only) presents analysis to identify which women are more likely to experience spousal violence and maternal health problems. Women’s lack education, husband’s lack education, poor wealth status and living in rural regions associate with both spousal violence and less than four ANC visits for most recent birth, no institutional delivery for last birth and terminated pregnancy.

## Discussion

We examined the trends in prevalence of spousal violence and maternal health by using the data from the two most recent demographic health surveys 2012–13 (PDHS) and 2017–18 (PDHS). Besides, we conducted predictive analysis on these datasets to determine the impact of spousal violence on maternal health. Findings show that there is some decline (2% to 5%) in rates of spousal violence and slight improvement (5% to 7%) on maternal health indicators over the years between these two surveys. However, this is not a satisfactory progress towards achieving goals of sustainable development and a raise serious concern about disparities in women’s access to maternal healthcare. 24% of women are still exposed to less severe physical violence; 51% of women had less than 4 ANC visits, 33% have not accessed institutional delivery and 32% had experienced terminated pregnancy. Current study findings consolidate the previous evidence that spousal violence significantly increases the risk of poor maternal health in women [34]. Findings of present analysis showed that women exposed to spousal violence are at increased risk for ‘ever having terminated pregnancy’ and having ‘two or more pregnancy losses.’ Findings from this analysis also showed that spousal violence significantly decreases the probability of antenatal visits during pregnancy. This finding aligns with previous research and has important implication in Pakistan’s context because one in every 10 women face violence during their pregnancy in low-income and middle-income countries [35].

This situation implies the need for more appropriate community health programs to outreach women and need to adopt more efficient ways to mobilize existing healthcare networks. The ground reality is that healthcare professionals are usually less sensitive and not trained to deal with spousal violence cases. Findings from a qualitative study showed that health care professionals are reluctant to inquire or screen women for spousal violence [36]. They consider it does not come under the domain of healthcare and its increases their already huge work burden. There is a need that healthcare professionals be motivated by offering appropriate financial incentives and support system to facilitate women who seek maternal healthcare services and facing problem of spousal violence. Besides the guidelines published by World Health Organization (WHO) can be used to establish supportive network and health services within existing healthcare system to pay special attention to the needs of women facing spousal violence [37].

According to WHO’s Focused Antenatal Care (FANC), there should be minimum of four antenatal visits for a pregnant woman without any complications (USAID, 2007). Current study findings are alarming, as 24% of women reported no antenatal visits. Current findings also suggest that women who face violence made less number of antenatal visits which aligns with previous literature [38]. Women who do not seek appropriate antenatal care become more vulnerable for poor pregnancy outcomes [39]. Screening and appropriate support services thus needs to be incorporated into community out-reach health programs as well as maternal healthcare centers [40, 41]. Findings suggest that government should allocate funds for community-based health programs and social interventions. Provision of good quality antenatal care and education about factors, which determine maternal health will improve the prospects of healthy lives for both mother and newborn.

Findings of present analysis showed that women exposed to spousal violence are less likely to seek institutional services for the delivery of last childbirth. Previous literature [42] though suggests that women, who experienced physical violence, were more at risk to be hospitalized due to pregnancy complications, pre-term labor, fetal distress and caesarean birth, however, association of emotional violence with maternal health outcomes has not been reported in existing literature [43]. Existing healthcare services should focus more on attending the greater need of intervention of women exposed to various forms of violence to prevent poorer maternal and neonatal outcomes.

### Strengths and limitations of study

The important strength of this research is that we used data from demographic health survey, which included nationally representative sample from both urban and rural areas of Pakistan and findings are generalizable to other developing countries of this region with similar healthcare system.

Among limitations, the survey did not have data on other indicators of women health such as maternal morbidity and mortality and not included in analysis. Secondly, keeping in view the social and cultural environment of Pakistani society, spousal violence is likely to be under-reported by participants in current survey. A large number of women participants were living in combined family systems and chances are high that they are reluctant to talk about experience of spousal violence.

### Implications of study

The impacts of spousal violence on maternal health are preventable through appropriate interventions, such as training health services staff in screening and early intervention in existing reproductive healthcare setting. The occurrence of spousal abuse is associated with number of social and cultural factors e.g. lack of economic independence, stigma and negative attitudes of people towards women who report abuse or seek help. There is need of increased political support and funding to implement variety of social, economic and health interventions. More outreach programs can ensure provision of antenatal care to pregnant women in the community. Psycho-behavioral interventions on family violence need to include educating families and husbands about repercussions of physical and emotional violence on mother health and child life prospects. Collective interventions at macro, meso and micro level in context of Pakistan and other developing countries are required to prevent spousal violence and improve indicators of maternal health.

## Conclusions

This study provides additional evidence about impacts of spousal violence on maternal health outcomes such as terminated pregnancy, deficient antenatal care and decreased likelihood of intuitional delivery. There is slight decrease in rates of spousal violence and slight improvement on maternal health indicators as indicated by the comparative analysis of 2012–13 and 2017–18 surveys. Nonetheless, this improvement is not substantial enough to achieve targets for sustainable development goals. These findings emphasize upon need for more integrative community interventions that should focus both on reducing spousal violence as well improving access to maternal health services in vulnerable populations.

## Supporting information

S1 Appendix (DOCX)
