# Supplementary material for: Utilization of maternal healthcare services in women experiencing spousal violence in Pakistan: A comparative analysis of 2012-13 and 2017-18 Pakistan Demographic Health Surveys
Source: PLoS One. 2020 Sep 25;15(9):e0239722. doi: 10.1371/journal.pone.0239722 (PMC7518579; doi:10.1371/journal.pone.0239722)
Supplement: S1 Appendix — (DOCX) [file pone.0239722.s001.docx]

**APPENDIX**

**Table 1** *Crosstab analysis between demographic variables, spousal violence and maternal health indicators for PHDS 2012-13*

|  | Less than 4 ANC visits & | | | | No Institutional Delivery for recent birth & | | | | Ever had terminated pregnancy & | | | |
| --- | --- | --- | --- | --- | --- | --- | --- | --- | --- | --- | --- | --- |
| Sociodemographic  Characteristics | **Less severe physical violence**  **(n=448; 36.5%)** | **Severe physical violence**  **(n=111; 9.1%)** | **Emotional violence (n=476; 38.9%)** | **Violence during pregnancy (n=194; 15.8)** | **Less severe physical violence**  **(n=360;38.3)** | **Severe physical violence**  **(n=89; 9.5%)**  **(360.;38.3)** | **Emoti-onal violence**  **(n=391;**  **41.6)** | **Violence during pregnancy**  **(n=158;**  **16.8%)** | **Less severe physical violence**  **(n=436; 32.9)** | **Severe physical violence**  **(n=116;8.8%)** | **Emotional violence**  **(n=471;35.5)** | **Violence during pregnancy (n=148;**  **11.2%)** |
|  | **Frequency (%)** | **Frequency (%)** | **Frequency (%)** | **Frequency (%)** | **Frequency (%)** | **Frequency (%)** | **Frequency (%)** | **Frequency (%)** | **Frequency (%)** | **Frequency (%)** | **Frequency (%)** | **Frequency (%)** |
|  | ns | ns | ns | ns | **ns** | **ns** | **ns** | **ns** | **ns** | **ns** | **ns** | **ns** |
| 15-19 | 10 (2.2%) | 4 (3.6%) | 10 (2.1%) | 6 (3.1%) | 7(1.9) | 3 (3.4) | 8 (2.0) | 13 (1.7) | 5 (1.1) | 3 (2.6) | 3 (0.6) | 1 (0.7) |
| 20-24 | 78 (17.4%) | 18 (16.2%) | 74 (15.5%) | 28 (14.4%) | 56(15.6) | 13 (14.6) | 55 (14.1) | 131 (16.8) | 36 (8.3) | 10 (8.6) | 39 (8.3%) | 7 (4.7) |
| 25-29 | 108 (24.1%) | 22 (19.8%) | 116 (24.4%) | 38 (19.6%) | 91 (25.3) | 16 (18.0) | 100(25.6) | 216 (27.7) | 66 (15.1) | 17 (14.7) | 65 (13.8) | 26 (17.6) |
| 30-34 | 117 (26.1%) | 29 (26.1%) | 129 (27.1%) | 54 (27.8%) | 99 (27.5) | 26 (29.2) | 108(27.6) | 212 (27.1) | 94 (21.6) | 22 (19.0) | 96 (20.4) | 33 (22.3) |
| 35-39 | 89 (19.9%) | 19 (17.1%) | 95 (20.0%) | 45 (23.2%) | 68 (18.9) | 16 (18.0) | 74(18.9) | 131 (16.8) | 92 (21.1) | 25 (21.6) | 104 (22.1) | 33 (23.3) |
| 40-44 | 35 (7.8%) | 14 (12.6%) | 41 (8.6%) | 20 (10.3%) | 27 (7.5) | 10 (11.2) | 34(8.7) | 49 (6.3) | 76 (17.4) | 16 (13.8) | 82 (17.4) | 21 (14.2) |
| 45-49 | 11 (2.5%) | 5 (4.5%) | 11 (2.3%) | 3 (1.5%) | 12 (3.3) | 5 (5.6) | 12 (3.1) | 29 (3.7) | 67 (15.4) | 23 (19.8) | 82 (17.4) | 27 (18.2) |
| Education | ns | ns | ns | ns | ns | p=.04 | ns | ns | p=.001 | p=.01 | p=.001 | p=.001 |
| No education | 335 (74.8) | 85 (76.6) | 350 (73.5) | 150(77.3) | 276 (76.7) | 75 (84.3) | 295(75.4) | 127 (8.4) | 290 (66.5) | 83 (71.6) | 309 (65.7) | 114 (77.0) |
| Primary | 34 (7.6) | 9(8.1) | 37 (7.8) | 23 (11.6) | 49 (13.6) | 11 (12.4) | 58 (14.8) | 16 (10.1) | 63 (14.4) | 14 (12.1) | 70 (14.9) | 14 (9.5) |
| Secondary | 17 (3.8) | 3 (2.7) | 22 (4.6) | 19 (9.3) | 32 (8.9) | 2 (2.2) | 34 (8.7) | 15 (9.5) | 58 (13.3) | 15 (12.9) | 53 (11.3) | 15 (10.1) |
| Higher | 9 (2.0) | 2 (1.8) | 12 (2.5) | 2 (1.0) | 3 (0.8) | 1 (1.1) | 4 (1.0) | 0 (0) | 25 (5.7) | 4 (3.4) | 38 (8.1) | 5 (3.4) |
| Women Current employment status* | p=.01 | ns | ns | ns | ns | ns | ns | ns | ns | ns | ns | ns |
| Unemployed | 617 (79.5) | 867(77.9) | 588(78.6) | 144(74.2) | 268(74.4) | 63 (70.8) | 288 (73.7) | 120 (75.9) | 313 (72.0) | 84 (72.4) | 339 (72.3) | 106 (71.6) |
| Employed | 159 (20.5) | 246(22.1) | 160(21.4) | 50 (25.8) | 92 (25.6) | 26 (29.2) | 103 (26.3) | 38 (24.1) | 123 (28.0) | 32 (27.6) | 130 (27.7) | 42 (28.4) |
| Husband Education | ns | p=.02 | p=.02 | ns | ns | p=.01 | p=.05 | ns | ns | ns | ns | ns |
| No education | 202(45.2) | 49(44.1) | 218(45.8) | 90(46.4) | 172 (47.9) | 46 (51.7) | 188 (48.1) | 79 (50.0) | 177 (40.7) | 51 (44.0) | 185 (39.4) | 69 (46.6) |
| Primary | 69 (15.1) | 23(21.1) | 69(13.5) | 30 (15.6) | 55 (15.3) | 18 (20.2) | 61 (15.6) | 25 (15.8) | 61 (14.0) | 20 (17.2) | 66(14.1) | 21 (14.2) |
| Secondary | 127 (28.2) | 28(25.2) | 134 (27.3) | 56(24.3) | 99 (27.6) | 18 (20.2) | 104 (26.6) | 41 (25.9) | 134 (30.8) | 36 (31.0) | 146 (31.1) | 41 (27.7) |
| Higher | 49 (11.0) | 10(9.0) | 55(11.6) | 18(9.3) | 33 (9.2) | 6 (6.7) | 38 (9.7) | 13 (8.2) | 62 (14.3) | 8 (6.9) | 70 (14.9) | 17 (11.5) |
| Husband Current employment status | ns | ns | ns | ns | ns | ns | ns | ns | ns | ns | ns | ns |
| Unemployed | 18 (4.0) | 5(4.5) | 17 (3.6) | 10 (5.2) | 11 (3.1) | 4(4.5) | 11 (2.8) | 8 (5.1) | 11 (2.5) | 3 (2.6) | 18 (3.8) | 4 (2.7) |
| Employed | 430 (96.0) | 106 (96.1) | 459 (97.1) | 184 (95.2) | 349 (97.1) | 85 (96.5) | 380 (98.2) | 150 (95.5) | 425 (98.2) | 113 (98.6) | 453 (97.3) | 144 (98.3) |
| Wealth Index | p=.01 | ns | ns | ns | ns | ns | ns | ns | p=.001 | ns | p=.001 | p=.001 |
| Poorest | 146(32.6) | 34(30.6) | 150(31.5) | 69(35.6) | 124 (34.4) | 36 (40.4) | 136 (34.8) | 64 (40.5) | 105 (24.1) | 31 (26.7) | 117 (24.9) | 51 (34.5) |
| Poorer | 131(29.2) | 29(26.1) | 146(30.7) | 53(27.3) | 96 (26.7) | 20 (22.5) | 114 (29.2) | 40 (25.3) | 84 (19.3) | 23 (19.8) | 89 (18.9) | 30 (20.3) |
| Middle | 101(22.5) | 30(27.0) | 93(19.5) | 44(22.7) | 78 (21.7) | 21 (23.6) | 77 (19.7) | 28 (17.7) | 81 (18.6) | 19 (16.4) | 83 (17.7) | 22 (14.9) |
| Richer | 53(11.8) | 12(10.8) | 67(14.1) | 23(11.9) | 47 (13.1) | 8 (9.0) | 49 (12.5) | 18 (11.4) | 96 (22.0) | 25 (21.6) | 108 (23.0) | 32 (21.6) |
| Richest | 17(3.8) | 6(5.4) | 20 (4.2) | 5 (7.8) | 15 (4.2) | 4 (4.5) | 15 (3.8) | 8 (5.1) | 70 (16.1) | 18 (15.5) | 73 (15.5) | 13 (8.8) |
| Place of Residence | ns | ns | p=.05 | ns | ns | ns | ns | ns | ns | ns | p=.001 | p=.001 |
| Urban | 140 (31.3) | 39 (35.1) | 135 (28.4) | 59 (30.4) | 120 (33.3) | 29 (32.6) | 118 (30.2) | 50 (31.6) | 179 (41.1) | 48 (41.4) | 186 (39.4) | 47 (31.8) |
| Rural | 308 (68.8) | 72 (64.9) | 341 (71.6) | 135 (69.6) | 240 (66.7) | 60 (67.4) | 273 (69.8) | 108 (68.4) | 257 (58.9) | 68 (58.6) | 285 (60.6) | 101 (68.2) |

**Table 2** *Crosstab analysis between demographic variables, spousal violence and maternal health indicators for PHDS 2017-18*

|  | Less than 4 ANC visits & | | | | | No Institutional Delivery for recent birth & | | | | | Ever had terminated pregnancy & | | | | |
| --- | --- | --- | --- | --- | --- | --- | --- | --- | --- | --- | --- | --- | --- | --- | --- |
| Sociodemographic  Characteristics | **Experienced less severe physical violence**  **(n=386;**  **33.4%)** | **Experienced severe physical violence**  **(n=106; 9.2%)** | **Experienced emotional violence (n=433; 37.4%)** | **Experienced sexual violence (n=65; 5.6%)** | **Experienced violence during pregnancy (n=113; 9.8)** | **Experienced less severe physical violence**  **(n=253;33.6)** | **Experienced severe physical violence**  **(n=72; 9.6%)** | **Experienced emotional violence**  **(n=284;**  **37.8)** | **Experienced sexual violence (n=39; 5.2%)** | **Experienced violence during pregnancy**  **(n=69;**  **9.2%)** | **Experienced less severe physical violence**  **(n=362; 27.6)** | **Experienced severe physical violence**  **(n=112;8.8%)** | **Experienced emotional violence**  **(n=421;32.1)** | **Experienced sexual violence (n=73; 5.6%)** | **Experienced violence during pregnancy (n=120;**  **9.1%)** |
|  | **Frequency (%)** | **Frequency (%)** | **Frequency (%)** | **Frequency (%)** | **Frequency (%)** | **Frequency (%)** | **Frequency (%)** | **Frequency (%)** | **Frequency (%)** | **Frequency (%)** | **Frequency (%)** | **Frequency (%)** | **Frequency (%)** | **Frequency (%)** | **Frequency (%)** |
|  | ns | ns | ns | ns | ns | ns | ns | ns | ns | ns | ns | ns | ns | ns | ns |
| 15-19 | 10 (2.6) | 3 (2.8) | 14 (3.2) | 2 (3.1) | 3 (2.7) | 8 (3.2) | 1 (1.4) | 9 (3.2) | 1 (2.6) | 2 (2.9) | 1 (0.3) | 0 (0.0) | 2 (0.5) | 0(0.0) | 0 (0.0) |
| 20-24 | 71 (18.4) | 16(15.1) | 69 (15.9) | 9 (13.8) | 25 (22.1) | 45(17.8) | 12(16.7) | 44(15.5) | 6 (15.4) | 13(18.8) | 34 (9.4) | 8 (7.1) | 30 (7.1) | 8 (11.0) | 10 (8.3) |
| 25-29 | 105 (27.2) | 28 (26.4) | 120 (27.7) | 17 (26.2) | 27 (23.9) | 63 (24.9) | 23 (31.9) | 75(26.4) | 9 (23.1) | 19 (27.5) | 68 (18.8) | 21 (18.8) | 67 (15.9) | 12 (16.4) | 15 (12.5) |
| 30-34 | 95 (24.6) | 30 (28.3) | 109 (25.2) | 18 (27.7) | 27 (23.9) | 57 (22.5) | 17 (23.6) | 68 (23.9) | 13 (33.3) | 17 (24.6) | 91 (25.1) | 27 (24.1) | 107 (25.4) | 18 (24.7) | 21 (25.8) |
| 35-39 | 74 (19.2) | 22 (20.8) | 81 (18.7) | 13 (20.0) | 19 (16.8) | 57 (22.5) | 14 (19.4) | 60 (21.1) | 6 (15.4) | 12 (17.4) | 81 (22.4) | 26 (23.2) | 104 (24.7) | 17 (23.3) | 32 (26.7) |
| 40-44 | 21 (5.4) | 6 (5.7) | 32 (7.4) | 4 (6.2) | 9 (8.0) | 14 (5.5) | 4 (5.6) | 20 (7.0) | 2 (5.1) | 4 (5.8) | 53 (14.6) | 18 (16.1) | 65 (15.4) | 10 (13.7) | 19 (15.8) |
| 45-49 | 10 (2.6) | 1 (0.9) | 8 (1.8) | 2 (3.1) | 3 (2.7) | 9 (3.6) | 1 (1.4) | 8 (2.8) | 2 (5.1) | 2 (2.9) | 34 (9.4) | 12 (10.7) | 46 (10.9) | 8 (11.0) | 13 (10.8) |
| Education | p=.001 | ns | p=.001 | ns | p=.04 | ns | ns | ns | p=.05 | p=.01 | p=.01 | p=.01 | p=.01 | p=.03 | p=.01 |
| No education | 299 (77.5) | 83 (78.3) | 334 (77.1) | 46 (7.8) | 89 (78.8) | 199 (78.7) | 57 (79.2) | 220 (77.5) | 29 (74.4) | 51 (73.9) | 220 (60.8) | 74 (66.1) | 244 (58.0) | 39 (53.4) | 74 (61.7) |
| Primary | 48 (12.4) | 14 (13.2) | 51 (11.8) | 13 (20.0) | 16 (14.2) | 28 (11.1) | 7 (9.7) | 33 (11.6) | 9 (23.1) | 15 (21.7) | 57 (15.7) | 17 (15.2) | 61 (14.5) | 18 (24.7) | 25 (20.8) |
| Secondary | 30 (7.8) | 7 (6.6) | 31 (7.2) | 5 (7.7) | 7 (6.2) | 21 (8.3) | 7 (9.7) | 22 (7.7) | 1 (2.6) | 3 (4.3) | 57 (15.7) | 14 (12.5) | 68 (16.2) | 11 (15.1) | 17 (14.2) |
| Higher | 9 (18.8) | 2 (1.9) | 17 (3.9) | 1 (1.5) | 1 (0.9) | 5 (2.0) | 1 (1.4) | 9 (3.2) | 0 (0.0) | 0 (0.0) | 28 (7.7) | 7 (6.3) | 48 (11.4) | 5 (6.8) | 4 (3.3) |
| Women Current employment status* | ns | ns | ns | p=.02 | p=.02 | p=.02 | ns | ns | ns | ns | ns | ns | ns | ns | ns |
| Unemployed | 346 (89.6) | 90 (84.9) | 388 (89.6) | 51 (78.5) | 100 (88.5) | 222(87.7) | 60 (83.3) | 248 (87.3) | 31 (79.5) | 59 (85.5) | 298 (82.3) | 87 (77.7) | 336 (79.8) | 59 (80.8) | 94 (78.3) |
| Employed | 40 (10.4) | 16 (15.1) | 45 (10.4) | 14 (21.5) | 13 (11.5) | 31 (12.3) | 12 (16.7) | 36 (12.7) | 8 (20.5) | 10 (14.5) | 64 (17.7) | 25 (22.3) | 85 (20.2) | 14 (19.2) | 26 (21.7) |
| Husband Education | ns | p=.02 | p=.02 | ns | ns | p=.01 | p=.05 | ns | ns | ns | ns | ns | ns | p=.02 | p=.02 |
| No education | 294 (76.1) | 83 (78.3) | 334 (77.1) | 46 (7.8) | 89 (78.8) | 199 (78.7) | 57 (79.2) | 220 (77.5) | 29 (74.4) | 51 (73.9) | 220 (60.8) | 74 (66.1) | 244 (58.0) | 39 (53.4) | 74 (61.7) |
| Primary | 54 (13.4) | 14 (13.2) | 51 (11.8) | 13 (20.0) | 16 (14.2) | 28 (11.1) | 7 (9.7) | 33 (11.6) | 9 (23.1) | 15 (21.7) | 57 (15.7) | 17 (15.2) | 61 (14.5) | 18 (24.7) | 25 (20.8) |
| Secondary | 28 (7.8) | 7 (6.6) | 31 (7.2) | 5 (7.7) | 7 (6.2) | 21 (8.3) | 7 (9.7) | 22 (7.7) | 1 (2.6) | 3 (4.3) | 57 (15.7) | 14 (12.5) | 68 (16.2) | 11 (15.1) | 17 (14.2) |
| Higher | 10 (18.8) | 2 (1.9) | 17 (3.9) | 1 (1.5) | 1 (0.9) | 5 (2.0) | 1 (1.4) | 9 (3.2) | 0 (0.0) | 0 (0.0) | 28 (7.7) | 7 (6.3) | 48 (11.4) | 5 (6.8) | 4 (3.3) |
| Husband Current employment status | ns | ns | ns | ns | ns | ns | ns | ns | ns | ns | ns | ns | ns | ns | ns |
| Unemployed | 38 (10.4) | 18 (15.1) | 44 (10.4) | 17 (21.5) | 18 (11.5) | 32 (12.3) | 10 (16.7) | 36 (12.7) | 8 (20.5) | 10 (14.5) | 64 (17.7) | 25 (22.3) | 85 (20.2) | 14 (19.2) | 26 (21.7) |
| Employed | 344 (89.6) | 92 (84.9) | 387 (89.6) | 53 (78.5) | 98 (88.5) | 221 (87.7) | 58 (83.3) | 248 (87.3) | 31 (79.5) | 59 (85.5) | 298 (82.3) | 87 (77.7) | 336 (79.8) | 59 (80.8) | 94 (78.3) |
| Wealth Index | ns | ns | ns | ns | ns | ns | ns | ns | ns | ns | p=.01 | p=.01 | p=.03 | p=.01 | p=.02 |
| Poorest | 153 (39.6) | 47 (44.3) | 168 (38.8) | 28 (43.1) | 47 (41.6) | 100 (39.5) | 32 (44.4) | 111 (39.1) | 15 (38.5) | 29 (42.0) | 127 (35.1) | 46 (41.1) | 132 (31.4) | 32 (43.8) | 45 (37.5) |
| Poorer | 109 (28.2) | 20 (18.9) | 116 (26.8) | 11 (16.9) | 26 (23.0) | 77 (30.4) | 17 (23.6) | 83 (29.2) | 11 (28.2) | 17 (24.6) | 74 (20.4) | 18 (16.1) | 88 (20.9) | 8 (11.0) | 24 (20.0) |
| Middle | 72 (18.7) | 28 (26.4) | 85 (19.6) | 15 (23.1) | 24 (21.2) | 49 (19.4) | 16 (22.2) | 57 (20.1) | 8 (20.5) | 15 (21.7) | 70 (19.3) | 24 (21.4) | 82 (19.5) | 12 (16.4) | 23 (19.2) |
| Richer | 30 (7.8) | 7 (6.6) | 40 (9.2) | 7 (10.8) | 11 (9.7) | 16 (6.3) | 5 (6.9) | 21 (7.4) | 4 (10.3) | 5 (7.2) | 51 (14.1) | 14 (12.5) | 60 (14.3) | 10 (13.7) | 18 (15.0) |
| Richest | 22 (5.7) | 4 (3.8) | 24 (5.5) | 4 (6.2) | 5 (4.4) | 11 (4.3) | 2 (2.8) | 12 (4.2) | 1 (2.6) | 3 (4.3) | 40 (11.0) | 10 (8.9) | 59 (14.0) | 11 (15.1) | 10 (8.3) |
| Place of Residence | ns | ns | p=.05 | ns | ns | ns | ns | ns | ns | ns | p=.001 | p=.001 | ns | ns | p=.05 |
| Urban | 118 (30.6) | 36 (34.0) | 121 (27.9) | 25 (38.5) | 35 (31.0) | 74 (29.2) | 26 (36.1) | 82 (28.9) | 16 (41.0) | 18 (26.1) | 159 (43.9) | 54 (48.2) | 187 (44.4) | 36 (49.3) | 51 (42.5) |
| Rural | 268 (69.4) | 70 (66.0) | 312 (72.1) | 40 (61.5) | 78 (69.0) | 179 (70.8) | 46 (63.9) | 202 (71.1) | 23 (59.0) | 51 (73.9) | 203 (56.1) | 58 (51.8) | 234 (55.6) | 37 (50.7) | 69 (57.5) |
